# Supplementary material for: 3D-Printed Collagen Scaffolds Promote Maintenance of Cryopreserved Patients-Derived Melanoma Explants
Source: Cells. 2021 Mar 7;10(3):589. doi: 10.3390/cells10030589 (PMC8000141; doi:10.3390/cells10030589)
Supplement: Supplementary file 1 [file cells-10-00589-s001.pdf]

3D-Printed Collagen Scaffolds Promote Maintenance of Cryopreserved Patients-Derived Melanoma Explants

Yun-Mi Jeong [1,*,§], ChulHwan Bang [2,*], MinJi Park[1], Sun Shin[3], Seokhwan Yun[1], Chul Mi Kim[1,5], GaHee Jeong [4], Yeun-Jun Chung[3], Woo-Soo Yun [1], Ji Hyun Lee [2,§] and Songwan Jin [1]

[1]Department of Mechanical Engineering, Korea Polytechnic University, Siheung-si, Gyoenggi-do, Korea, 15073

[2]Department of Dermatology, Seoul St. Mary's Hospital, College of Medicine, The Catholic University of Korea, Seoul, Korea

[3]Precision Medicine Research Center, IRCGP, Department of Biomedicine & Health Sciences, Graduate School, The Catholic University of Korea, College of Medicine. Seoul, Korea

[4]Department of Biomedicine & Health Sciences, The Catholic University, Seoul, Korea

[5]Department of Mechatronics, Gyeongsang National University, JinJu, Gyeongsangnam-do, Korea

Correspondence: [1]phdjeongym12@kpu.ac.kr and [2]ejee@catholic.ac.kr;
Tel.: [1]+82-31-8041-1819, [2]+82-2-599-9950

*co-first and co-corresponding authors contributed equally to this work.

Methods

Melanoma patients

We enrolled two melanoma patients with one nodular melanoma on the back and one lentigo malignant melanoma on the chin (Fig. S1A). Informed consent was received from the patients prior to enrollment. This study protocol was reviewed by the Institutional Review Boards of the Seoul St. Mary's Hospital (KC15TISI0966). Table S1 in Fig. S1 shows the patients' information, including surgical and pathology information. Wide excision was performed on the two patients, and melanoma tissues for PDX were acquired at the time of surgery. In particular, patient #2 melanoma tissues for PDX were collected from the newly expanded area.

Measurement of a palpable tumor volume in the MPDX models

To establish MPDX models, our criteria were: A) the first day of engrafted PDXs were zero days. B) left side and - right side of tumor volume endpoint in MPDX model was not exceed 2000 mm^3. Body weight (g) were measured using an electronic balance (model GR-200 and CUX220H) and left side/right side length width was measured at the indicated days. The mass size of a palpable tumor volume in the MPDX models = tumor maximum length $\times$ $width^2/2$.

A

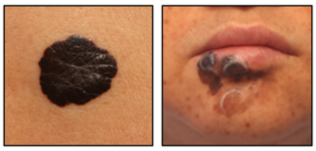

Case	Age	Sex	Primary site	Type	Surgery	TNM	Stage
Patient 1	62	M	Back	Nodular	Wide excision and local fla p coverage	T1aN0M0	Stage IA
Patient 2	59	M	Chin	Lentigo maligna melanoma	Wide excision and Bernard burrow flap coverage	T1aN0M0	Stage IA

B

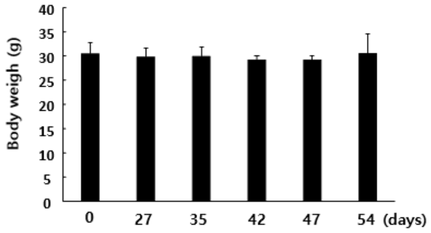

Fig. S1. Information on the two melanoma patients studied and body weight of the MPDX models at the indicated days. (A) Clinical photography of patient 1 (upper left) and patient 2 (upper right). Table 1(lower) shows the patients' information, including surgical and pathology. (B) Body weight of MPDX models.

A

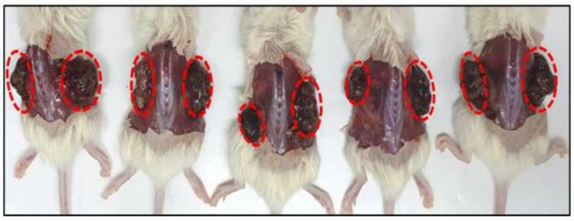

B

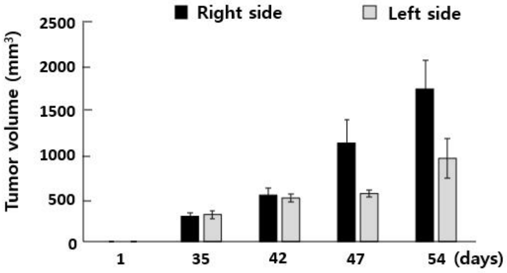

Fig. S2. Left - and right side of tumor volume endpoint in MPDX model. (A) Images of a MPDX model after euthanasia. At the tumor volume endpoint 54 days, the mice were euthanized. Red circle dots show tumor volume of left - and right side. (B) Graphs indicated growth of tumor volume (mm^3) at the indicated days.

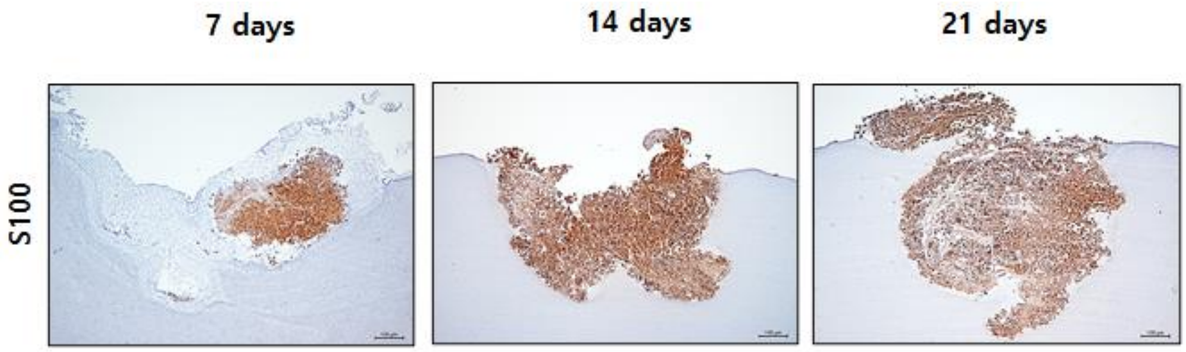

Fig. S3. Expression of S100 in cryopreserved PDME cultured onto the 3D-printed collagen scaffolds. IHC analysis for maintaining the function of melanoma marker S100 at the indicated days.
